# Pan-genome wide identification and analysis of the *SAMS* gene family in sunflowers (*Helianthus annuus* L.) revealed their intraspecies diversity and potential roles in abiotic stress tolerance

**DOI:** 10.3389/fpls.2024.1499024

**Published:** 2024-11-13

**Authors:** Chun Zhang, Haoyu Li, Jiamin Yin, Zhibin Han, Xinqi Liu, Yang Chen

**Affiliations:** ^1^ Department of Agronomy, Hetao College, Bayannur, China; ^2^ Bayannur Modern Agriculture and Animal Husbandry Development Center, Bayannur, China

**Keywords:** S-adenosylmethionine synthase, pan-genome, sunflowers, abiotic stresses, qRT-PCR

## Abstract

**Introduction:**

S-adenosylmethionine (SAM), a key molecule in plant biology, plays an essential role in stress response and growth regulation. Despite its importance, the SAM synthetase *(SAMS)* gene family in sunflowers *(Helianthus annuus L.)* remains poorly understood.

**Methods:**

In this study, the *SAMS* genes were identified from the sunflower genome. Subsequently, the protein properties, gene structure, chromosomal location, cis-acting elements, collinearity, and phylogeny of the SAMS gene family were analyzed by bioinformatic methods. Finally, the expression patterns of *SAMS* genes in different tissues, under different hormonal treatment and abiotic stress were analyzed based on transcriptome data and qRT-PCR.

**Results:**

This study identified 58 SAMS genes across nine cultivated sunflower species, which were phylogenetically classified into seven distinct subgroups. Physicochemical properties and gene structure analysis showed that the *SAMS* genes are tightly conserved between cultivars. Collinearity analysis revealed segmental duplications as the primary driver of gene family expansion. The codon usage bias analysis suggested that natural selection substantially shapes the codon usage patterns of sunflower *SAMS* genes, with a bias for G/C-ending high-frequency codons, particularly encoding glycine, leucine, and arginine. Analysis of the cis-regulatory elements in promoter regions, implied their potential roles in stress responsiveness. Differential expression patterns for HanSAMS genes were observed in different tissues as well as under hormone treatment or abiotic stress conditions by analyzing RNA-seq data from previous studies and qRT-PCR data in our current study. The majority of genes demonstrated a robust response to BRA and IAA treatments in leaf tissues, with no significant expression change observed in roots, suggesting the response of *HanSAMS* genes to hormones is tissue-specific. Expression analyses under abiotic stresses demonstrated diverse expression profiles of *HanSAMS* genes, with *HanSAMS5* showing significant upregulation in response to both drought and salt stresses.

**Discussion:**

This comprehensive genomic and expression analysis provides valuable insights into the *SAMS* gene family in sunflowers, laying a robust foundation for future functional studies and applications in crop improvement for stress resilience.

## 1 Introduction

Plants have developed sophisticated and adaptable mechanisms to adjust to challenging environments, involving a spectrum of morphological, physiological, and molecular changes (Ahuja et al., 2010). They frequently employ strategies such as strengthening and preserving the integrity of biological membranes, along with boosting the production of antioxidant enzymes, to endure stresses from cold, drought, and high salinity (Yang et al., 2013; Mehari et al., 2021). The cultivated sunflower (*Helianthus annuus* L.) is a prominent oil crop with global significance, renowned for its resilience in adverse environmental conditions,which is originally domesticated by Native Americans in North America, and later introduced to Europe and subsequently became a vital crop worldwide (Zukovsky, 1950; Mantenese et al., 2006). Nevertheless, the cultivation of sunflowers faces various challenges, with drought and salinity being prominent abiotic stressors (Rele and Mohile, 2003; Keeley et al., 2021). A multitude of gene families, including S-adenosyl-L-methionine synthase (SAMS), are integral to the complex regulatory networks that dictate plant stress responses, impacting their growth and bolstering their resilience to harsh conditions (He et al., 2019).

*SAMS* genes are distinguished by the presence of a methionine-binding site in their N-terminal domain and an ATP-binding motif in their C-terminal domain. These enzymes catalyze the formation of SAM (S-Adenosyl-L-methionine) through the condensation of methionine with ATP, playing a crucial role in essential biological pathways within eukaryotic cells (Heidari et al., 2020). Numerous *SAMS* genes have been identified by researchers (Ahuja et al., 2010; Yang et al., 2013; Heidari et al., 2020). In *Arabidopsis thaliana*, there exist four *SAMS* genes, with *AtSAMS3* demonstrating predominant expression within pollen tissues (Yang et al., 2013). The suppression of *OsSAMS1*, 2, and 3 in rice (*Oryza sativa*) led to alterations in histone modifications and DNA methylation patterns, which in turn triggered a delay in flowering time (Li et al., 2011). Espartero et al. observed that the expression of *SAMS* in tomatoes (*Solanum lycopersicum*) was altered in response to salt stress (Heidari et al., 2020). Similarly, in cucumbers (*Cucumis sativus*), salt stress induced the expression of *SAMS*, implicating its role in the modulation of associated stress-response mechanisms (Roje, 2006; Bürstenbinder et al., 2007). In soybean (*Glycine max*), the expression profiles of the *SAMS* gene family exhibited significant variation in the face of drought and waterlogging stress, yet displayed relative stability under treatments involving sodium chloride (NaCl) and low temperatures (Jang et al., 2012; Ma et al., 2017). The gene *GhSAMS2* has emerged as a promising candidate for the genetic enhancement of upland cotton’s resistance to multiple abiotic stresses (Gupta et al., 2013). The overexpression of *CsSAMS1* and its interaction with *CsCDPK6* resulted in the stimulation of ethylene and polyamines biosynthesis, ultimately improving salt stress tolerance in transgenic tobacco (*Nicotiana tabacum*) plants (Zhu et al., 2021). Overexpressing *Medicago sativa subsp. Falcata SAMS1* in transgenic tobacco plants increased their tolerance to cold stress by enhancing oxidation and polyamine synthesis (Guo et al., 2014).

Pan-genomic analysis, now a prevalent approach, is utilized to assess genetic variability within species, explore gene flow between species, and examine the processes of domestication and crop improvement (Hübner et al., 2019; Li et al., 2010; Gao et al., 2019; Tao et al., 2021; Tettelin et al., 2005). A single reference genome might not capture the full spectrum of genetic diversity that evolves over time within a species, possibly leading to the exclusion of many important genes. While the *SAMS* gene family has been extensively researched in *A. thaliana*, rice, cotton, and tomato, there is a pronounced shortfall in studies on the *SAMS* genes in sunflowers, particularly in relation to their pan-genome diversity and how their expression patterns react to abiotic stresses such as cold, drought, and salinity, as well as to external hormonal signals (He et al., 2019; Heidari et al., 2020; Sun et al., 2022).

In this research, we conducted a comprehensive, genome-wide identification of *SAMS* genes utilizing the sunflower pan-genome. A total of 58 *SAMS* genes were discovered across nine cultivated sunflower varieties. We investigated their phylogenetic relationships, gene structures, motifs, *cis*-elements, and the secondary and tertiary structures of the corresponding proteins. Additionally, we analyzed the codon usage bias in these 58 *SAMS* genes, employing neutrality plot, ENc-plot, PR2-plot, and the Relative Synonymous Codon Usage (RSCU) method. Building on this, we extracted gene expression data for the *SAMS* gene from a variety of conditions, including exposure to abiotic stresses and treatments with external hormones. Furthermore, we performed a systematic analysis of the *SAMS* gene expressions, with a particular focus on their expression patterns under drought and salt stress conditions, using quantitative real-time PCR (qRT-PCR). These results provided comprehensive genomic information of sunflower *SAMS* gene family, enhancing our understanding of their roles in stress response and potentially contributing to the development of sunflower varieties with improved stress tolerance.

## 2 Materials and methods

### 2.1 Identification of *SAMS* genes

Protein sequences of SAMSs from *A. thaliana* were obtained from the TAIR (https://www.Arabidopsis.org/). Genome and annotation files of *Helianthus annuus* XRQ) was downloaded from Ensembl plants (https://plants.ensembl.org/index.html). *Helianthus annuus* (HA89), *Helianthus annuus* (HA300), *Helianthus annuus* (IR), *Helianthus annuus* (LR1), *Helianthus annuus* (OQP8), *Helianthus annuus* (PI659440), *Helianthus annuus* (PSC8) and *Helianthus annuus* (RHA438) were downloaded from NCBI. The Hidden Markov Model (HMM) (PF02772, PF02773, PF00438) of S-adenosylmethionine synthase was downloaded from the Pfam database (https://pfam.xfam.org/), and were used to search protein databases by HMMER in TBtools-II (Chen et al., 2023) with an E-value<1e−5. Subsequently, all putative *SAMS* genes shared the three HMM domains were validated by batch-CD search (http://www.ncbi.nlm.nih.gov/Structure/cdd/wrpsb.cgi), Pfam, and HMMER (https://www.ebi.ac.uk/Tools/hmmer/) databases (Potter et al., 2018; Mistry et al., 2021; Wang et al., 2023). The *SAMS* genes in the XRQ cultivar are named using Latin abbreviations coupled with their chromosomal positions in the XRQ reference genome. For instance, the designation *XRQ-HanSAMS1* indicates that XRQ represents the cultivar name, Han refers to Helianthus annuus, and the numeral in SAMS corresponds to the gene’s ordered position on the chromosome, listed from the smallest to the largest. Other cultivars’ genes keep their names but get a SAMS number based on where they group with *XRQ-HanSAMS* genes in the evolution tree. Furthermore, the biochemical parameters of HanSAMS were determined using the ProtParam tool (https://web.expasy.org/protparam/) (Gasteiger et al., 2005). Finally, the subcellular localizations of *HanSAMS* were predicted using the WoLF PSORT (https://wolfpsort.hgc.jp/). The NPS@: SOPMA secondary structure (https://npsa-prabi.ibcp.fr/cgi-bin/npsa_automat.pl?page=npsa_sopma.html) was used to predict the secondary structures of HanSAMS proteins. SWISS-MODEL (https://swissmodel.expasy.org/) was employed to 3D protein structure prediction and PyMOL software was used to draw 3D structures of SAMS proteins (PyMOL molecular graphics system; http://www.pymol.org) (DeLano, 2002).

### 2.2 Phylogenetic, gene structure, *cis*-element, motif and collinear analysis

Multiple sequences alignments of the full-length SAMS protein sequences was performed using the ClustalX (Larkin et al., 2007). The Neighbor-joining (NJ) tree was constructed by MEGA7.0 with the amino acid substitution Poisson model and 1000 bootstrap replicates test model (Kumar et al., 2016). The exon-intron structure of the *SAMS* genes was analysed using GSDS 2.0 (http://gsds.cbi.pku.edu.cn/) (Hu et al., 2015). Conserved domains of SAMS proteins were analysed by MEME (http://meme.sdsc.edu/meme/cgi-bin/meme.cgi). The upstream 2000 bp sequences relative to the start codon of each *SAMS* gene were obtained to analyze the promoter regions, and the *cis*-elements within these regions were predicted using the PlantCARE (http://bioinformatics.psb.ugent.be/webtools/plantcare/html/) (Lescot et al., 2002). We employed BLASTP to identify homologous genes, with key parameters set to an e-value threshold of 1e-3 and a maximum of 10 target sequences. To identify collinear genes using MCScanX (Wang et al., 2012), we applied the default parameters, include an E_VALUE of 1e-05 and a MAX_GAPS count of 25. The nonsynonymous substitution rate/synonymous substitution rate (Ka/Ks) values were calculated via the DnaSP 6.0 application released by Universitat de Barcelona.

### 2.3 Estimation of codon bias

A Python-compiled custom program was used to calculate the genomic composition of the *SAMS* gene family across nine cultivated sunflower varieties, determining the total GC content (GCall) as well as the GC content at the first (GC1), second (GC2), the average GC content at the first and second positions (GC12) and third (GC3) codon positions within the coding DNA sequences (CDS). Additionally, we utilized the software CodonW v1.4.4 (http://codonw.sourceforge.net) to assess the relative synonymous codon usage (RSCU), count the number of effective codons (ENc), and calculate the codon adaptation index (CAI), also determining the length of the amino acid sequences. Furthermore, we conducted a series of analyses to visualize the codon usage bias and neutrality: the Neutrality plot, the PR2 plot, the ENc-plot, and the RSCU plot were all generated using R software.

### 2.4 Analysis of RNA-seq data of *HanSAMS*

Hormonal response expression data (NCBI accession number SRP092742) were sourced from the SunExpress V1 database, which provides a comprehensive resource for exploring the expression patterns of genes under various conditions in sunflowers. The FPKM values for all *XRQ-HanSAMS* genes were extracted and subsequently processed using TBtools-II to create heatmaps.

### 2.5 Plant cultivation, treatments, RNA isolation, and qRT-PCR

The sunflower salt-tolerant inbred line 19S05 was used to explore the influence of salt and drought stress on sunflower seedlings. We sowed high-quality sunflower seeds in a perforated plastic container filled with nutrient-rich soil, ensuring they received regular watering every three days to support their healthy development. The plants were grown under a controlled photoperiod of 16 hours of light followed by 8 hours of darkness, all within a stable room temperature range of 21 to 25 degrees Celsius(Song et al., 2024b). Once the sunflower seedlings reached the four-true-leaf stage, seedings were treated with 150 mM NaCl solution and 15% PEG6000 solution, respectively. The leaves were then collected at 0 h, 1 h, 3 h, 6 h, 12 h, and 24 h, immediately frozen in liquid nitrogen, and stored at −80°C. The total RNA isolation and purification of samples were performed using an RNAprep Pure Plant Plus Kit (rich in polysaccharides and polyphenolics) (Tiangen, Beijing, China). The RNA isolation for gene expression was done in biological replicates for each sample analyzed. RNA integrity was visualized by 1% agarose gel electrophoresis. The concentration and purity of RNAs (OD260/OD280>1.95) were determined with a NanoDrop Onemicrovolume UVvis spectrophotometer (NanoDrop Technologies, DE, USA). Further, 1 ug of total RNA was reverse transcribed in a 20 ul reaction volume using a PrimeScript RT reagent kit with a gDNA eraser (Code No.6210A, Takara, Beijing, China) following the manufacturer’s instructions to remove traces of contaminant DNA and prepare cDNA. 1 µg of purified total RNA was reverse transcribed into the first strand cDNA that was used to qRT-PCR. Quantitative real-time polymerase chain reaction (qRT-PCR) analysis was used to analyze the expression level of the identified *HanSAMSs*. The standard qRT-PCR with SYBR Premix Ex Taq II (TaKaRa, Beijing, China) was repeated at least three times on a CFX96 real-time System (BioRad, Beijing). Subsequently, Cycling parameters were 95°C for 30 s, 40 cycles of 95°C for 5 s, and 60°C for 30 s. For melting curve analysis, a program including 95°C for 15 s, followed by a constant increase from 60°C to 95°C, was included following the PCR cycles. Primer Premier 6.0 software were used to designed the specific primers of *HanSAMS* genes according to their gene sequences, listed in **Supplementary Table S1**. Results were analyzed by the 2^−△△Ct^ method using the *HanActin* as the endogenous reference gene (He et al., 2019).

## 3 Results

### 3.1 Pangenome-wide identification of *SAMS* gene family in sunflowers

A total of 58 SAMS genes are identified based on the nine sunflowers genomes, including 7 *XRQ-HanSAMS*, 6 *HA89-HanSAMS*, 7 *HA300-HanSAMS*, 6 *IR-HanSAMS*, 7 *LR1-HanSAMS*, 6 *PI659440-HanSAMS*, 6 *PSC8-HanSAMS*, 6 *OQP8-HanSAMS*, and 7 *RHA438-HanSAMS* (**Table 1**). The physicochemical properties of the *SAMS* genes were presented in **Table 1**. Their protein sequence length ranged from 390 to 391 aa, with a molecular weight (MW) varying from 42583.22 to 43012.92 Da. The isoelectric points (pI) of the protein ranged from 5.47 to 5.97. The grand average of hydropathicity (GRAVY) of the proteins ranged from -0.291 to -0.357, all were the hydrophobic proteins. Secondary structure prediction analysis revealed that the proteins encoded by all the genes were predominantly composed of α-helices, β-turns, random coils, and extended chains (**Supplementary Table S2**). Among these, random coils were the most abundant structural element, accounting for 40.26% to 45.9% of the secondary structure. α-helices were the next most common, representing a proportion of 30.51% to 37.69%. Extended chains followed with a composition of 13.85% to 16.92%. The least prevalent structure was β-turns, which constituted only 6.92% to 8.97% of the total secondary structure content. Tertiary structure prediction showed that seven SAMS proteins from the reference genome XRQ were matching prediction s-adenosylmethionine synthase 2, which including 2 diphosphomethylphosphonic acid adenosyl ester and 2 potassium ion (**Supplementary Figure S1**).

**Table 1:** The information of the identified HanSAMS gene family in nine sunflowers.

Gene Name	Gene ID	Chr	Start	End	Number of amino acids (aa)	Molecular weight (Da)	Theoretical pI	Grand average of hydropathicity (GRAVY)
*XRQ-HanSAMS1*	HanXRQr2_Chr01g0040721	Chr01	141178594	141179766	390	42968.88	5.97	-0.346
*XRQ-HanSAMS2*	HanXRQr2_Chr02g0076781	Chr02	151379397	151381775	390	42667.34	5.67	-0.337
*XRQ-HanSAMS3*	HanXRQr2_Chr05g0218761	Chr05	123974636	123977188	391	42737.51	5.65	-0.291
*XRQ-HanSAMS4*	HanXRQr2_Chr07g0301771	Chr07	118073833	118076445	390	42637.31	5.58	-0.319
*XRQ-HanSAMS5*	HanXRQr2_Chr11g0515381	Chr11	179525398	179526763	390	42768.54	5.86	-0.341
*XRQ-HanSAMS6*	HanXRQr2_Chr13g0586041	Chr13	83908346	83910875	390	42583.22	5.73	-0.316
*XRQ-HanSAMS7*	HanXRQr2_Chr14g0659171	Chr14	154860282	154862804	390	42640.32	5.58	-0.303
*HA89-HanSAMS1*	HanHA89Chr01g0035721	Chr01	141952252	141953424	390	42968.88	5.97	-0.346
*HA89-HanSAMS2*	HanHA89Chr02g0072371	Chr02	151050725	151053081	390	42667.34	5.67	-0.337
*HA89-HanSAMS3*	HanHA89Chr05g0193641	Chr05	123509230	123511775	391	42737.51	5.65	-0.291
*HA89-HanSAMS4*	HanHA89Chr07g0265261	Chr07	118227621	118230059	390	42637.31	5.58	-0.319
*HA89-HanSAMS6*	HanHA89Chr13g0512461	Chr13	83922587	83925120	390	42583.22	5.73	-0.316
*HA89-HanSAMS7*	HanHA89Chr14g0584821	Chr14	156049129	156051559	390	42640.32	5.58	-0.303
*HA300-HanSAMS1*	HanHA300Chr01g0033191	Chr01	138801146	138802318	390	43012.92	5.97	-0.344
*HA300-HanSAMS2*	HanHA300Chr02g0063931	Chr02	148706034	148708390	390	42667.34	5.67	-0.337
*HA300-HanSAMS3*	HanHA300Chr05g0178901	Chr05	117579400	117581945	391	42737.51	5.65	-0.291
*HA300-HanSAMS4*	HanHA300Chr07g0248451	Chr07	115405490	115407929	390	42637.31	5.58	-0.319
*HA300-HanSAMS5*	HanHA300Chr11g0422801	Chr11	174480957	174482129	390	42768.54	5.86	-0.341
*HA300-HanSAMS6*	HanHA300Chr13g0480381	Chr13	82039590	82042123	390	42583.22	5.73	-0.316
*HA300-HanSAMS7*	HanHA300Chr14g0536951	Chr14	144837421	144839851	390	42640.32	5.58	-0.303
*IR-HanSAMS2*	HanIRChr02g0089631	Chr02	151201584	151206312	390	42667.34	5.67	-0.337
*IR-HanSAMS3*	HanIRChr05g0235181	Chr05	126120008	126122677	391	42737.51	5.65	-0.291
*IR-HanSAMS4*	HanIRChr07g0325441	Chr07	118275055	118277458	390	42637.31	5.58	-0.319
*IR-HanSAMS5*	HanIRChr11g0553961	Chr11	179753853	179758008	390	42768.54	5.86	-0.341
*IR-HanSAMS6*	HanIRChr13g0638071	Chr13	82142037	82144570	390	42583.22	5.73	-0.316
*IR-HanSAMS7*	HanIRChr14g0715241	Chr14	158625950	158628504	390	42640.32	5.58	-0.303
*LR1-HanSAMS1.1*	HanLR1Chr00c0365g0744971	–	50299	51471	390	42968.88	5.97	-0.346
*LR1-HanSAMS1.2*	HanLR1Chr00c0566g0760211	–	47071	48243	390	43012.92	5.97	-0.344
*LR1-HanSAMS2*	HanLR1Chr02g0066821	Chr02	151075783	151076955	390	42667.34	5.67	-0.337
*LR1-HanSAMS4*	HanLR1Chr07g0247581	Chr07	117507373	117508545	390	42637.31	5.58	-0.319
*LR1-HanSAMS5*	HanLR1Chr11g0424191	Chr11	179222692	179223864	390	42768.54	5.86	-0.341
*LR1-HanSAMS6*	HanLR1Chr13g0482441	Chr13	81444379	81445551	390	42594.16	5.47	-0.311
*LR1-HanSAMS7*	HanLR1Chr14g0547181	Chr14	157346616	157349011	390	42638.3	5.58	-0.318
*OQP8-HanSAMS1*	HanOQP8Chr01g0034171	Chr01	167373744	167374916	390	42952.82	5.97	-0.353
*OQP8-HanSAMS2*	HanOQP8Chr02g0077651	Chr02	166481855	166484211	390	42667.34	5.67	-0.337
*OQP8-HanSAMS4*	HanOQP8Chr07g0255101	Chr07	117648767	117651187	390	42637.31	5.58	-0.319
*OQP8-HanSAMS5*	HanOQP8Chr11g0424921	Chr11	177950410	177951582	390	42768.54	5.86	-0.341
*OQP8-HanSAMS6*	HanOQP8Chr13g0481321	Chr13	83037810	83040343	390	42583.22	5.73	-0.316
*OQP8-HanSAMS7*	HanOQP8Chr14g0544331	Chr14	155795294	155797687	390	42640.32	5.58	-0.303
*PI659440-HanSAMS1*	HanPI659440Chr00c05g0713751	–	1573106	1574278	390	42972.81	5.97	-0.357
*PI659440-HanSAMS2*	HanPI659440Chr02g0085791	Chr02	157900681	157906226	390	42667.34	5.67	-0.337
*PI659440-HanSAMS3*	HanPI659440Chr05g0204041	Chr05	125469449	125472052	391	42737.51	5.65	-0.291
*PI659440-HanSAMS5*	HanPI659440Chr11g0438411	Chr11	185595937	185599063	390	42768.54	5.86	-0.341
*PI659440-HanSAMS6*	HanPI659440Chr13g0489401	Chr13	28919635	28922154	390	42608.23	5.55	-0.312
*PI659440-HanSAMS7*	HanPI659440Chr14g0565851	Chr14	148426212	148428354	390	42640.32	5.58	-0.303
*PSC8-HanSAMS1*	HanPSC8Chr01g0039531	Chr01	147977048	147980576	390	42952.82	5.97	-0.353
*PSC8-HanSAMS2*	HanPSC8Chr02g0074491	Chr02	156100025	156102667	390	42667.34	5.67	-0.337
*PSC8-HanSAMS3*	HanPSC8Chr05g0211181	Chr05	124940843	124943410	391	42737.51	5.65	-0.291
*PSC8-HanSAMS4*	HanPSC8Chr07g0292091	Chr07	117662755	117665348	390	42637.31	5.58	-0.319
*PSC8-HanSAMS5*	HanPSC8Chr11g0496671	Chr11	179223032	179225152	390	42784.58	5.86	-0.327
*PSC8-HanSAMS7*	HanPSC8Chr14g0632221	Chr14	161860094	161862616	390	42640.32	5.58	-0.303
*RHA438-HanSAMS1*	HanRHA438Chr01g0041581	Chr01	144767362	144769020	390	42968.88	5.97	-0.346
*RHA438-HanSAMS2*	HanRHA438Chr02g0088081	Chr02	153746776	153749342	390	42667.34	5.67	-0.337
*RHA438-HanSAMS3*	HanRHA438Chr05g0227851	Chr05	123778849	123781427	391	42737.51	5.65	-0.291
*RHA438-HanSAMS4*	HanRHA438Chr07g0311691	Chr07	118251355	118254279	390	42637.31	5.58	-0.319
*RHA438-HanSAMS5*	HanRHA438Chr11g0527231	Chr11	178138655	178140891	390	42784.58	5.86	-0.327
*RHA438-HanSAMS6*	HanRHA438Chr13g0596681	Chr13	82615728	82618267	390	42583.22	5.73	-0.316
*RHA438-HanSAMS7*	HanRHA438Chr14g0670101	Chr14	155470650	155474584	390	42640.32	5.58	-0.303

### 3.2 Phylogenetic and evolution analysis of *HanSAMS* gene

**Figure 1:**
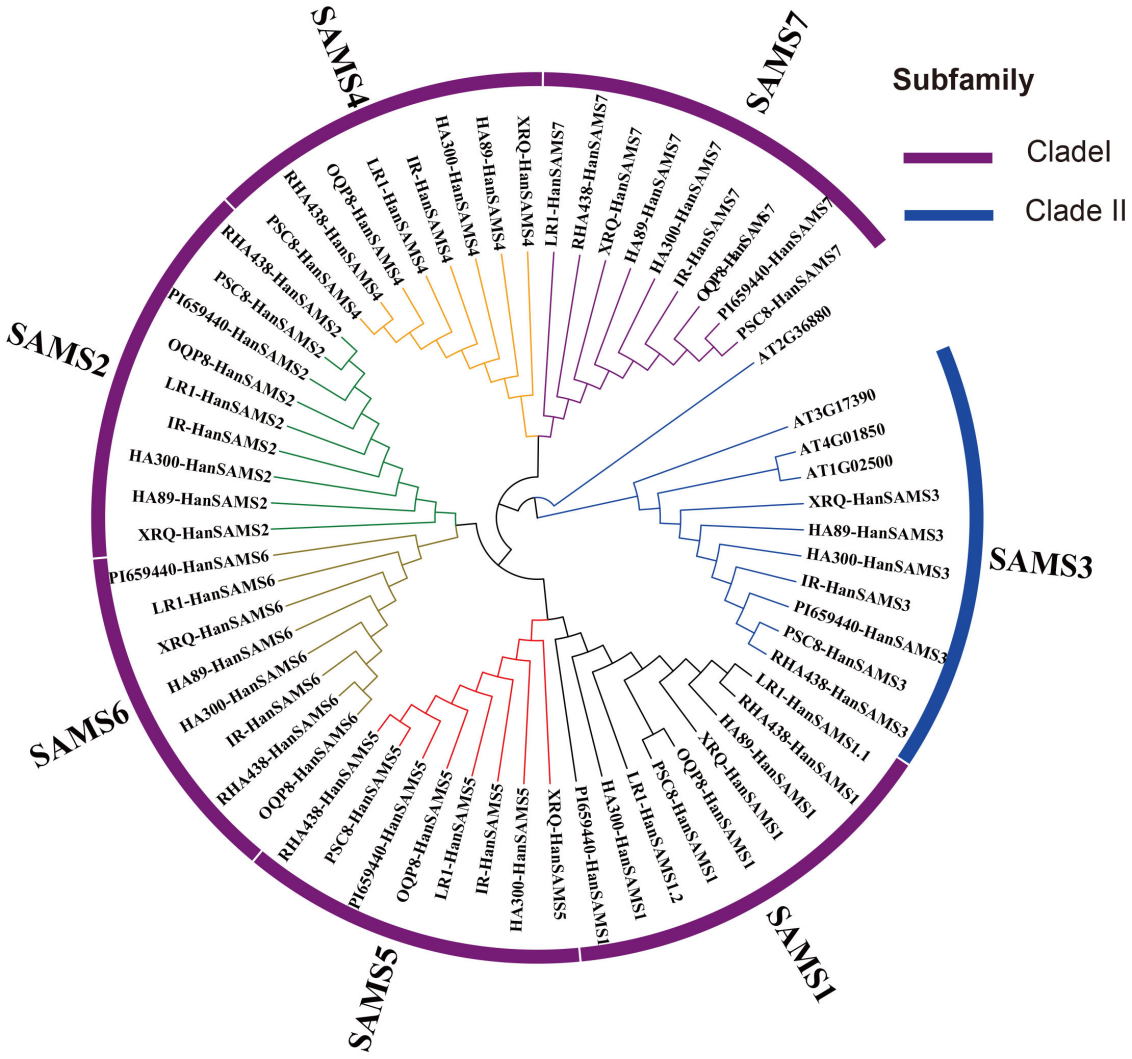
Identification of *HanSAMS* genes in nine cultivated sunflowers. Phylogenetic tree of nine cultivated sunflowers and Arabidopsis *SAMS* genes.

**Figure 2:**
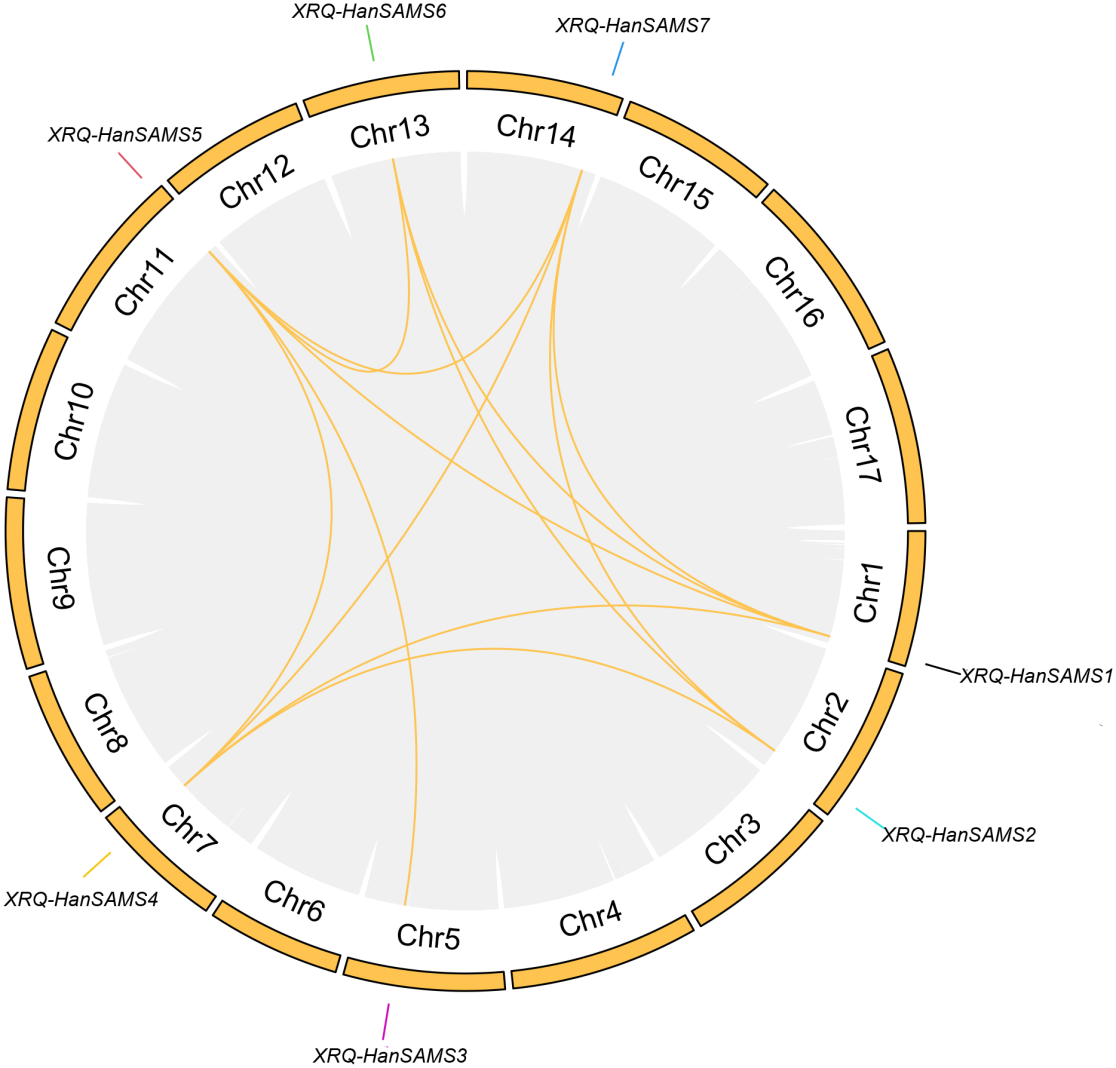
Chromosomal distribution and collinear relationships of the *HanSAMS* family. The collinearity of all genes within the sunflower is connected by gray background lines, and the collinearity where the SAMS gene is located is marked with yellow lines.

**Figure 3:**
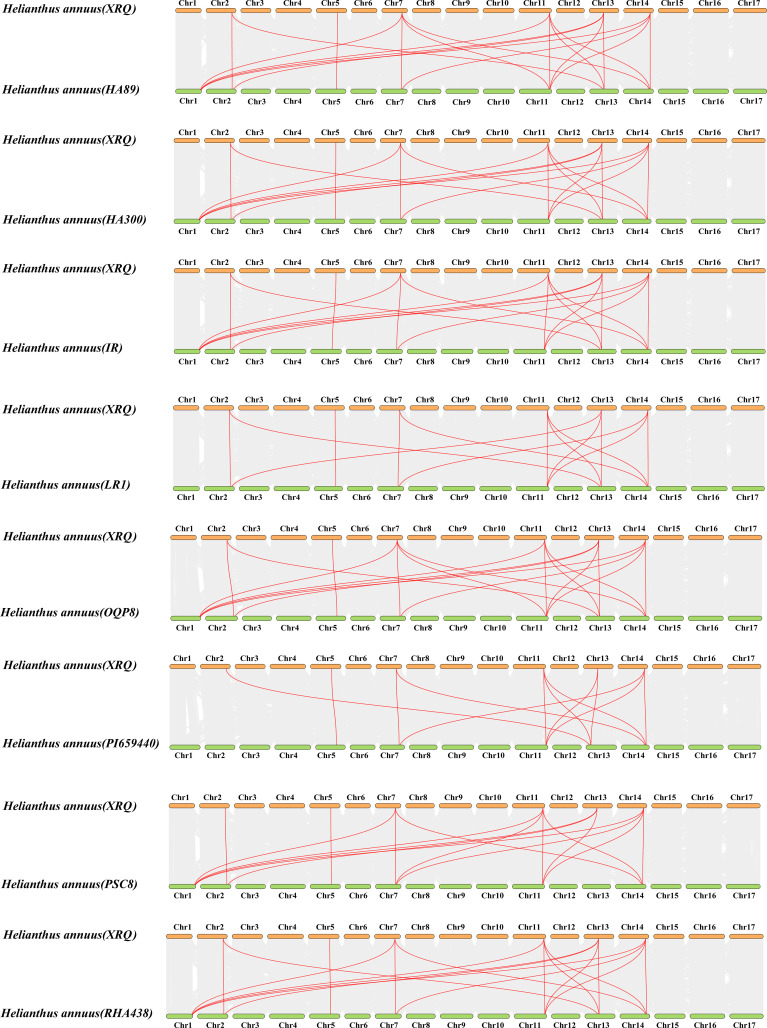
Collinearity analysis of *HanSAMS* gene families between *XRQ* and other eight cultivated sunflowers. gray lines indicate all synteny blocks in the sunflower genome, and the red lines indicate duplicated *SAMS* gene pairs, the chromosome number is indicated at the top or bottom of each chromosome.

The phylogenetic analysis of nine cultivated sunflower SAMS proteins were performed to examine their relationships. Based on the constructed phylogenetic tree, the *SAMS* genes could be classified into two major clades with seven groups (SAMS1-SAMS7) (**Figure 1**). Clade I, which is specific to sunflower *SAMS* genes, was found to branch into three main divisions, with each division containing a pair of distinct *SAMS* genes: SAMS1 with SAMS5, SAMS6 with SAMS2, and SAMS4 with SAMS7 (**Figure 1**). It is proposed that the WGT-1 event around 38-50 million years ago (Badouin et al., 2017) was possibly responsible for generating three homologs within the sunflower *SAMS* gene family, establishing the three principal branches. Subsequently, the WGD-2 event, occurring approximately 29 million years ago (Badouin et al., 2017), is believed to have caused the duplication of each branch, resulting in two copies per branch and shaping the present structure of the clade I gene family. Notably, the clade II only consists of the SAMS3 group and is uniquely distributed on a separate branch and forms a striking cluster with three homologs from *A. thaliana*. This finding suggests that the SAMS3 group may share a common ancient ancestor with *A. thaliana* and appears to have not undergone the most recent whole-genome duplication event, due to lacking the partnered SAMS group that are found clustered together in other SAMS groups. To explore the expansion mechanism of the *HanSAMS* gene family, we analyzed gene duplication events in sunflowers using the reference genome XRQ. We found that the *HanSAMS* genes are distributed across seven chromosomes, with one gene per chromosome (see **Figure 2**), and no tandem clusters were identified. Subsequently, we investigated the gene collinearity within sunflowers and identified 12 pairs of duplicated genes (**Figure 2**), suggesting that whole genome duplication (WGD) is the primary driver behind the expansion of the *HanSAMS* gene family. The Ka/Ks values were all lower than 1 for the duplicated genes (**Table 2**), indicated that the *SAMS* gene family in sunflower has predominantly experienced purifying selection. The interspecies collinearity analysis of the *HanSAMS* gene families among XRQ and other eight sunflowers was further performed, and it was found that there were 134 pairs of collinearity, including 19 pairs of HA89, 19 pairs of OQP8, 18 pairs of HA300, 18 pairs of IR, 18 pairs of RHA438, 16 pairs of PSC8, 14 pairs of LR1 and 12 pairs of PI659440 (**Figure 3**). The collinear relationship among HA89, OQP8 and XRQ genes is the strongest, followed by HA300, IR, RHA438, and the least in PI659440, which may reflect the divergency among nine different cultivated sunflowers.

**Table 2:** Ka Ks analysis of *HanSAMS* duplicated genes in XRQ genome.

Seq 1	Seq 2	Ka	Ks	Ka/Ks
*XRQ-HanSAMS1*	*XRQ-HanSAMS2*	0.044	1.530	0.029
*XRQ-HanSAMS1*	*XRQ-HanSAMS6*	0.052	1.196	0.043
*XRQ-HanSAMS2*	*XRQ-HanSAMS6*	0.013	0.625	0.021
*XRQ-HanSAMS2*	*XRQ-HanSAMS3*	0.085	2.911	0.029
*XRQ-HanSAMS4*	*XRQ-HanSAMS7*	0.009	0.504	0.018
*XRQ-HanSAMS4*	*XRQ-HanSAMS5*	0.034	1.088	0.031
*XRQ-HanSAMS4*	*XRQ-HanSAMS1*	0.051	2.056	0.025
*XRQ-HanSAMS4*	*XRQ-HanSAMS2*	0.033	1.448	0.023
*XRQ-HanSAMS4*	*XRQ-HanSAMS6*	0.038	1.724	0.022
*XRQ-HanSAMS4*	*XRQ-HanSAMS3*	0.069	4.345	0.016
*XRQ-HanSAMS5*	*XRQ-HanSAMS1*	0.031	0.817	0.038
*XRQ-HanSAMS5*	*XRQ-HanSAMS2*	0.027	1.233	0.022
*XRQ-HanSAMS5*	*XRQ-HanSAMS6*	0.030	1.166	0.025
*XRQ-HanSAMS6*	*XRQ-HanSAMS3*	0.095	2.458	0.039
*XRQ-HanSAMS7*	*XRQ-HanSAMS5*	0.038	1.347	0.028
*XRQ-HanSAMS7*	*XRQ-HanSAMS1*	0.052	2.098	0.025
*XRQ-HanSAMS7*	*XRQ-HanSAMS2*	0.033	1.303	0.025
*XRQ-HanSAMS7*	*XRQ-HanSAMS6*	0.038	1.486	0.025
*XRQ-HanSAMS7*	*XRQ-HanSAMS3*	0.073	1.725	0.042
Average		0.044	1.635	0.028

### 3.3 Genes structure and subcellular localization analysis

**Figure 4:**
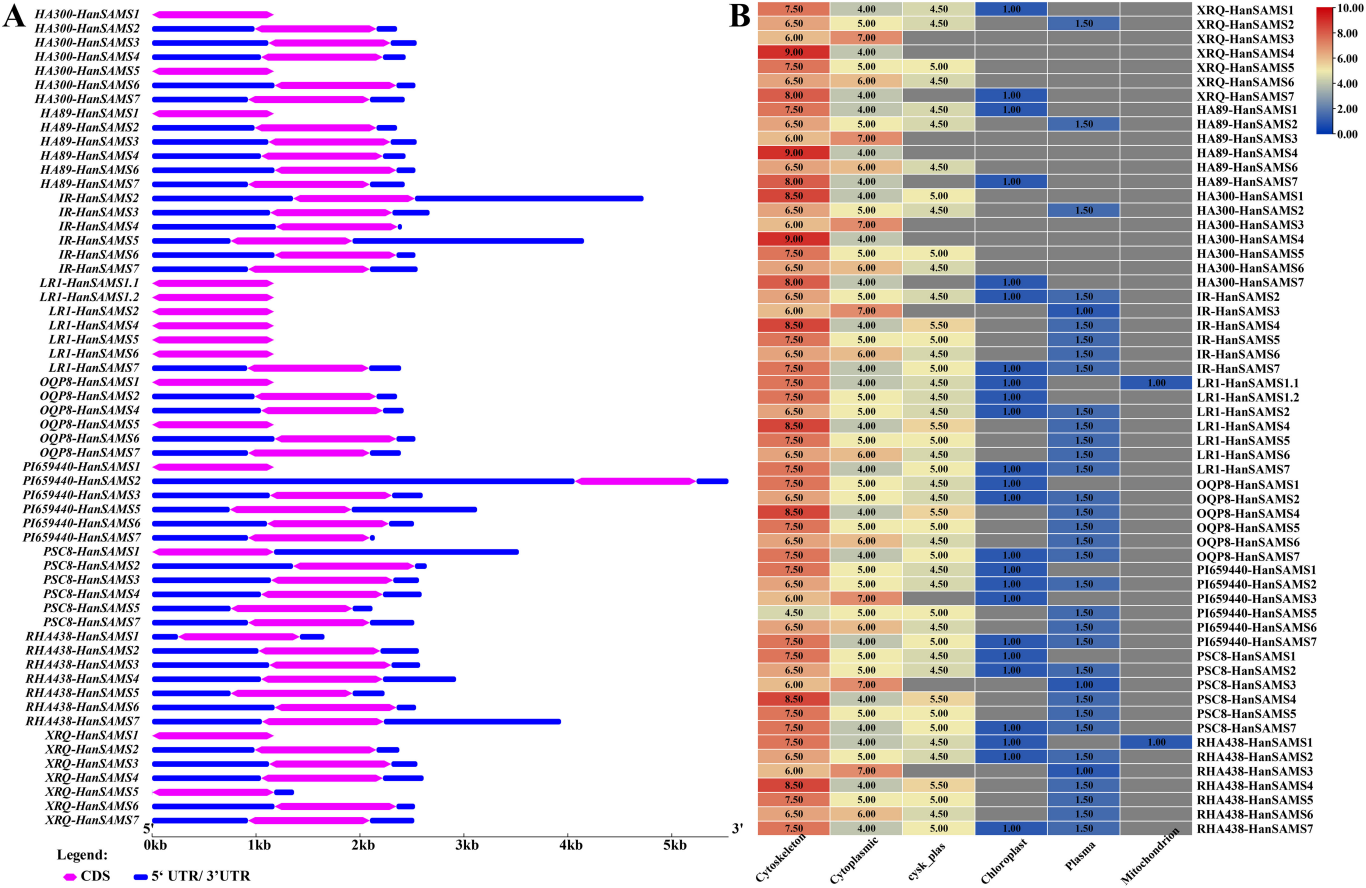
Gene structure and subcellular localization of *HanSAMS* genes in nine cultivated sunflowers. **(A)** Comparison of the gene structures of *SAMS* genes in nine cultivated sunflowers. **(B)** Comparison of subcellular localization of *HanSAMS* genes in nine cultivated sunflowers. The values in heatmap represents sorting signals for each candidate locations. Cysk_Plas, cytoskeleton and plasma membrane.

Examination of gene structures revealed that all *SAMS* gene in nine sunflowers have only one exon and are devoid of introns (**Figure 4A**). The *HA300-HanSAMS1*, *HA300-HanSAMS5*, *HA89-HanSAMS1*, *LR1-HanSAMS1.1*, *LR1-HanSAMS1.2*, *LR1-HanSAMS2*, *LR1-HanSAMS4*, *LR1-HanSAMS5*, *LR1-HanSAMS6*, *OQP8-HanSAMS1*, *OQP8-HanSAMS5*, *PI659440-HanSAMS1*, and *XRQ-HanSAMS1* were all lacked 5’ and 3’ untranslated regions (UTRs). The subcellular localization prediction suggested that the majority of SAMS proteins are predominantly found in the cytoskeleton, with the exception of SAMS3, which is localized in the cytoplasm, as shown in **Figure 4B**. The motifs of SAMS protein sequences were predicted using the MEME server, and all members of the *SAMS* contain motif1-motif10 (**Supplementary Figure S2A**), indicating highly conserved between different SAMS and different cultivars. The motif2 and motif5 were s-adenosylmethionine synthase domain (central domain), motif1 was s-adenosylmethionine synthase domain (N-terminal domain), motif3 and motif4 were s-adenosylmethionine synthase domain (C-terminal domain), about 50 amino acid residues long and is considered a key element (**Supplementary Figure S2B**).

### 3.4 Analysis of the codon usage bias of *HanSAMS* genes

The codon usage bias (CUB) of *SAM*S gene family in nine sunflowers species was investigated by analyzing the GC, GC1, GC2, and GC3 content (**Supplementary Table S3**). The GC content of the *HanSAMS* genes among the nine sunflower genomes ranged from 48.95% to 52.59%, with all group of *SAMS* and group SAMS3 having the lowest value 50% (**Table 3**). The GC1 content of all *HanSAMS* genes and the GC3 content of 91.38% of *HanSAMS* genes across nine sunflower species exceeded 50%, while the GC2 content remained below 50%. This suggests a notable variation in base composition at different positions and a pronounced bias towards G/C-rich start and stop codons. Although CUB across all *HanSAMS* genes was generally weak, as indicated by ENc values ranging from 41.76 to 53.62, there were variations among different SAMS groups. Specifically, HanSAMS2, HanSAMS4, and HanSAMS7 exhibited lower ENc values (<50%), suggesting a stronger preference for certain codons compared to HanSAMS groups 1, 3, 5, and 6 (see **Supplementary Table S3** for details). The ENc-plots of *HanSAMS* genes exhibit deviations from the expected curve, suggesting that natural selection predominantly influences CUB (**Supplementary Figure S3**).

**Table 3:** Average GC content and ENC values of *HanSAMS* genes in nine sunflowers.

Group	GC	GC1s	GC2s	GC3s	GC12	ENC
*HanSAMS1*	49.60	56.98	40.21	51.59	48.60	53.09
*HanSAMS2*	51.65	58.70	41.33	54.93	50.01	48.88
*HanSAMS3*	49.05	57.18	40.00	49.96	48.59	51.74
*HanSAMS4*	51.19	57.91	40.54	55.12	49.23	45.19
*HanSAMS5*	52.48	57.65	40.74	59.06	49.19	51.30
*HanSAMS6*	51.13	57.52	41.33	54.56	49.43	52.53
*HanSAMS7*	51.05	58.21	40.04	54.93	49.12	42.23

**Figure 5:**
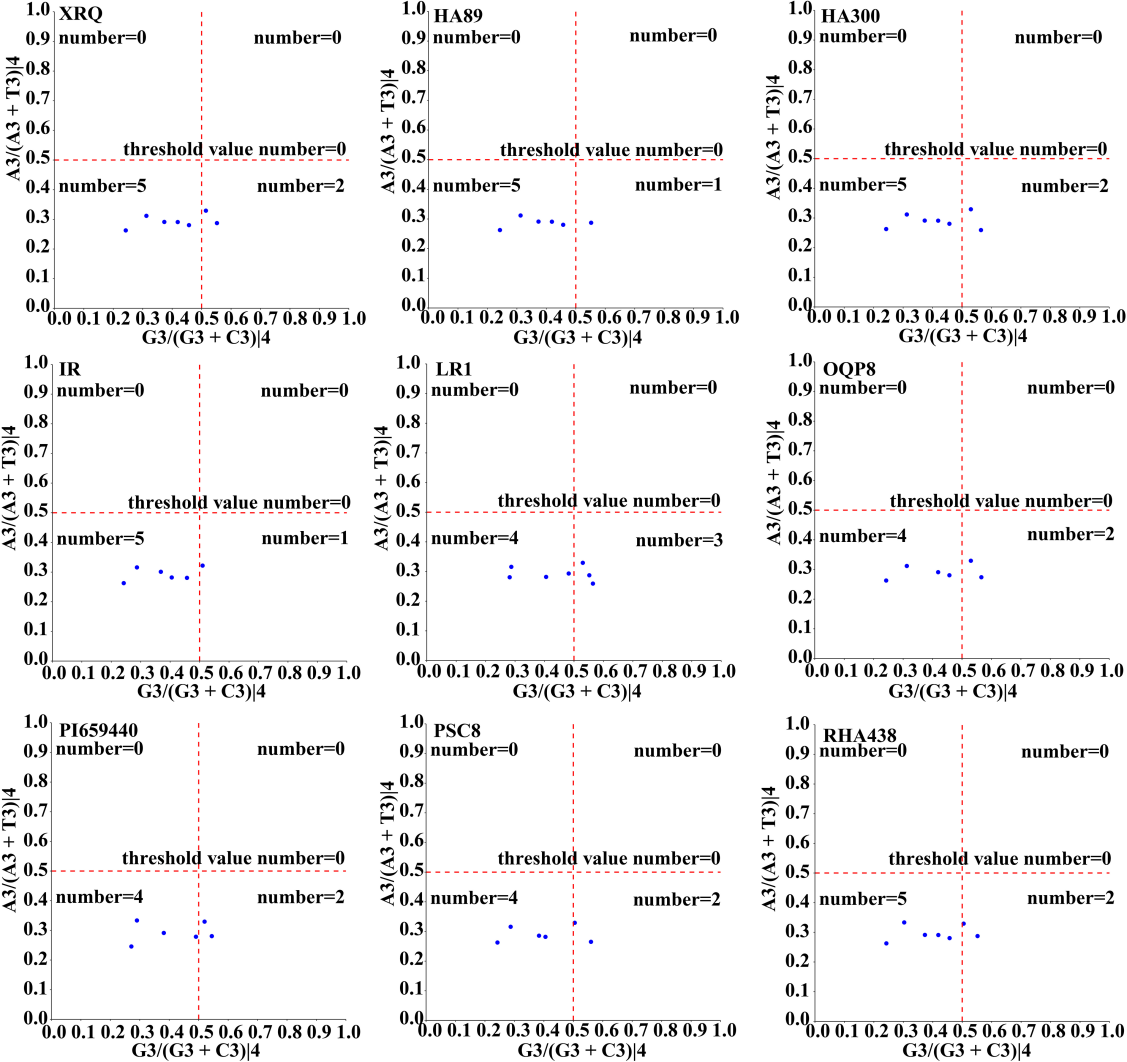
PR2-plot analysis of the *HanSAMS* gene family in nine sunflowers. A3/(A3+T3)|4 and G3/(G3+C3)|4 represents the four-codon degenerate amino acids.

The neutrality curve analysis of GC12 and GC3 values of nine sunflowers *HanSAMS* gene family revealed positive correlation between GC12 and GC3, with R values ranging from 0.11(IR-HanSAMS) to 0.72(HA89-HanSAMS) and the regression coefficients varying from 0.0576(IR-HanSAMS) to 0.18(HA89-HanSAMS), indicated that CUB of *HansSAMS* genes was mainly affected by natural selection (**Supplementary Figure S4**). The PR2-plot analysis reveals the distribution of the third base at the codon. The results show an uneven distribution of scatters across the four regions (**Figure 5**). Scatters in the top and bottom are predominantly in the lower half, indicating a preference for T at the third position. Those on the left and right are mostly in the left half, indicating a preference for C at the third position. Comparison among the quadrants shows the highest number of scatters in the quadrant three, suggesting a preference for C/T at the third position, implying that natural selection is the primary factor leading to CUB.

**Figure 6:**
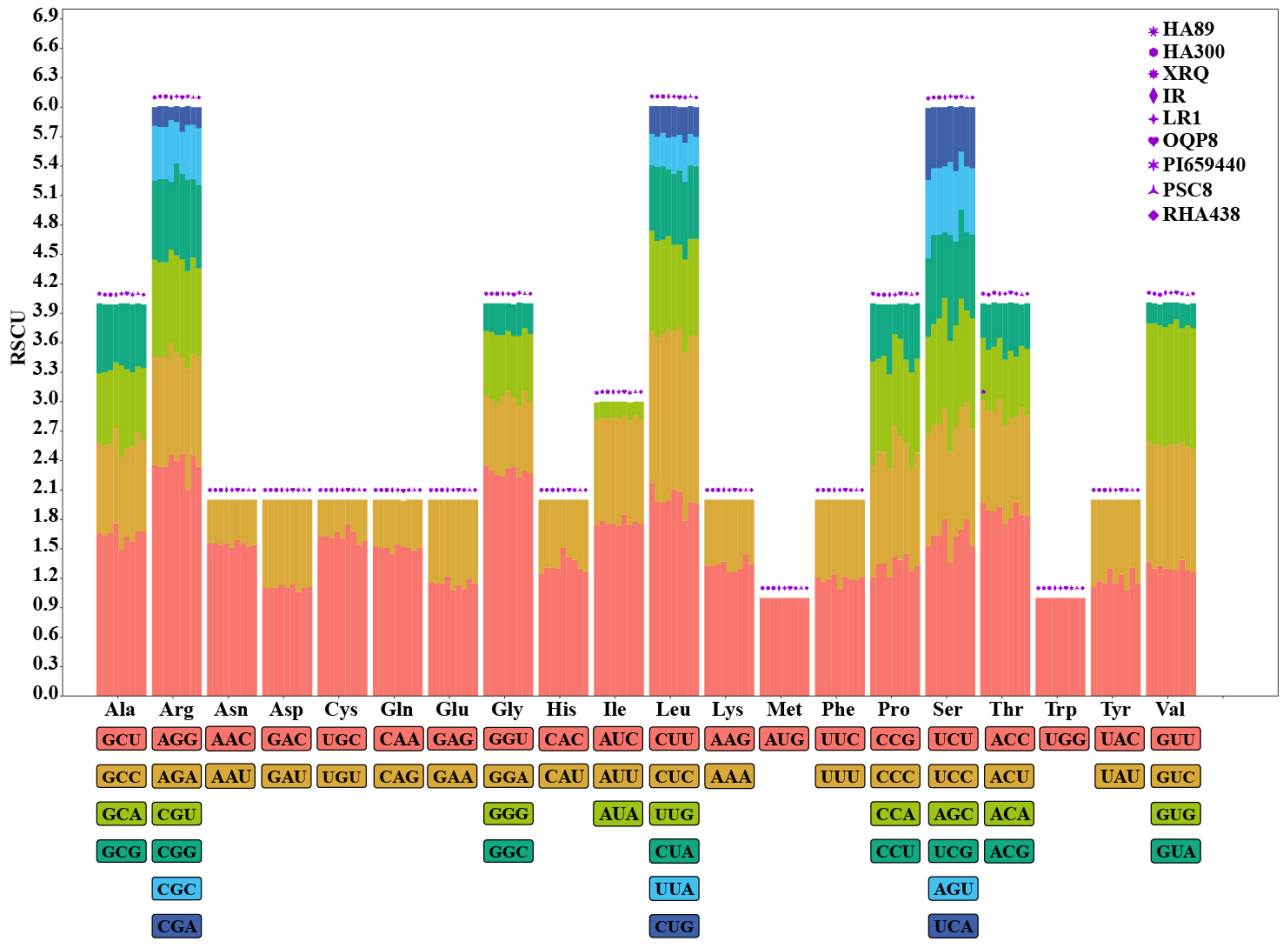
RSCU analysis of the *HanSAMS* gene family in nine cultivated sunflower species.

RSCU (relative synonymous codon usage) is a pivotal metric that quantifies CUB by comparing the observed frequency of each synonymous codon to its expected frequency under equal usage. The RSCU values of the *HanSAMS* genes were calculated and the results showed there were 22 codons shared by all nine sunflower cultivars with RSCU values greater than 1, of which 15 codons end with C/G (**Supplementary Table S4**; **Figure 6**). Conversely, low-frequency codons, which end in A/U, were also prevalent (22 of 35), indicating a bias for these codons in the gene family. The top 3 codons with the largest average RSCU value were encode Arg (AGG with RSCU 2.36), Gly (GGU with RSCU 2.29) and Leu (CUU with RSCU 2.00). The RSCU value varied among cultivars but were generally similar, suggesting a consistent pattern of codon usage across the *SAMS* gene family.

### 3.5 *Cis*-element analysis

**Figure 7:**
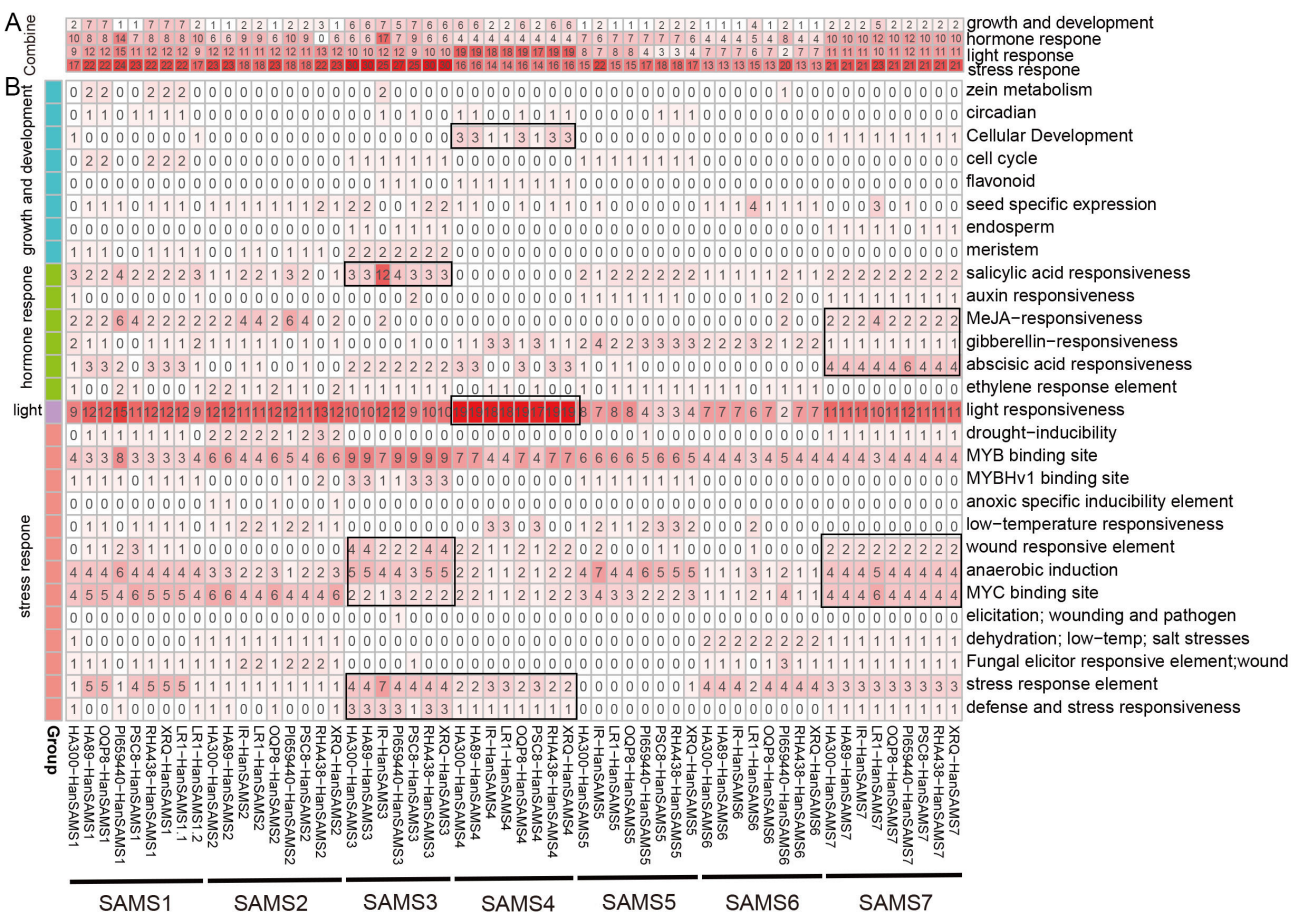
Analysis of the cis-element of *HanSAMS* genes. **(A)** Classification of cis-elements of *HanSAMS* promoters into four main groups: growth and development, hormone response, light responses and stress response. **(B)** Detail analysis of cis-elements in four groups for each *HanSAMS* gene promoter. The color intensity and number in each square indicate the number of each type of cis-element in the promoter region of the indicated gene. The distribution patterns of genes within the SAMS group that we are mentioned are marked with black boxes.

Promoter *cis*-acting elements are crucial for regulating gene expression. We utilized PlantCARE to identify *cis*-acting elements in the promoter regions of 58 *HanSAMS* genes (**Supplementary Table S5**, **Supplementary Figure S5**). Our statistical analysis showed these elements involved in various plant processes, including growth, development, hormone response, light response, and stress response (**Figure 7A**). Notably, stress response elements predominated in the *HanSAMS* promoters (**Figure 7A**). In detail analysis, 302 MYB binding site, 191 anaerobic induction (ARE element),186 MYC binding site and 149 stress response element (STRE element) were predicted with high frequency in the promoter region of *HanSAMS* genes (**Figure 7B**; **Supplementary Table S5**). Additionally, 624 light response-related elements were identified, such as MRE (n=105), GT1-motif (n=103), G-box (n=94), Box 4 (n=87) (**Supplementary Table S5**). Hormone response-related *cis*-regulatory elements were also observed, such as 108 salicylic acid responsiveness (TCA, as-1), 92 abscisic acid responsiveness (ABRE), 78 gibberellin-responsiveness, 74 MeJA-responsiveness (CGTCA-motif, TGACG-motif), and 41 ethylene response elements (ERE) (**Figure 7B**; **Supplementary Table S5**). We also noted that while most *SAMS* genes within the same group shared similar element distributions across different cultivars, some cultivars exhibited distinct differences (see marked with black boxes in **Figure 7B**). For instance, the *SAMS3* gene in the IR cultivar contained 12 salicylic acid response elements, which is significantly higher than other cultivars by at least three folds. These differences may be linked to the cultivars’ adaptability to environmental stresses and functional selection during evolution. Collectively, our findings suggest that *SAMS* genes are likely broadly involved in the regulation of hormones and stress responses.

### 3.6 Expression patterns of *HanSAMS* genes in different tissues

**Figure 8:**
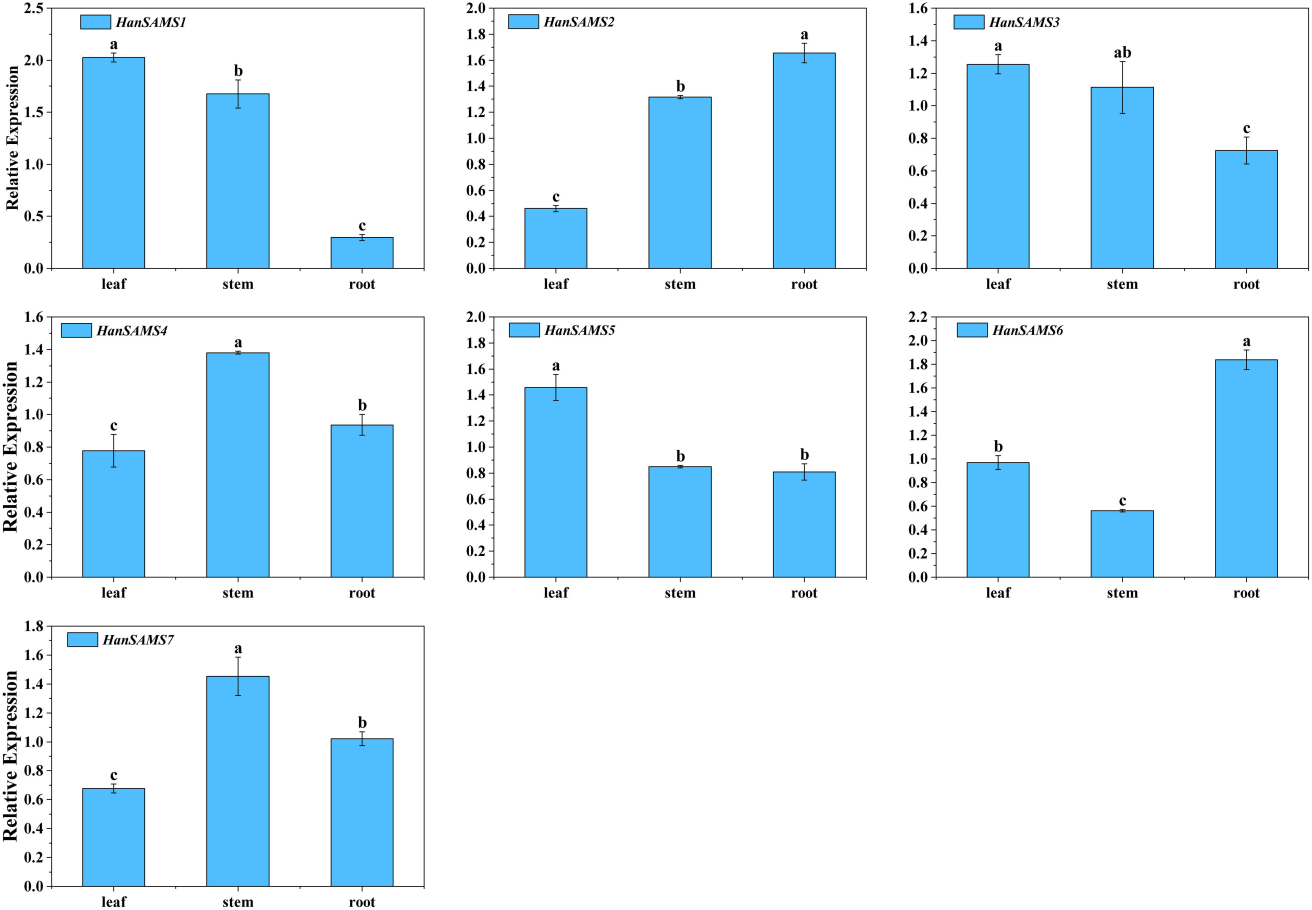
Expression profiles of *HanSAMS* genes in leaf, stem and root. a, b, c, bar indicates a significant difference among the different tissues (Significant differences were determined using the Duncan’s method of univariate ANOVA with a significance level of *P <0.05*).

*HanSAMS* genes may have different functions in the growth and development of sunflowers. To determine the spatial expression pattern of *HanSAMS* genes in sunflowers, we measured the expression levels of seven *XQR-HanSAMS* genes from three tissues (roots, stems, leaves) using qRT-PCR. As shown in **Figure 8**, seven *HanSAMS* genes were expressed in all the tissues. Among them, *HanSAMS1*, *HanSAMS3* and *HanSAMS5* had similar expression patterns and were expressed highest in leaves. Meanwhile, the expression of *HanSAMS4* and *HanSAMS7* was higher in stems than in the other three tissues. *HanSAMS2* and *HanSAMS6*, exhibited relatively high expression levels in the root. The results suggested that these genes showed a tissue-specific expression pattern and may play different roles in the growth and development of sunflowers.

### 3.7 Expression analysis of *HanSAMS* genes under different hormonal treatment

**Figure 9:**
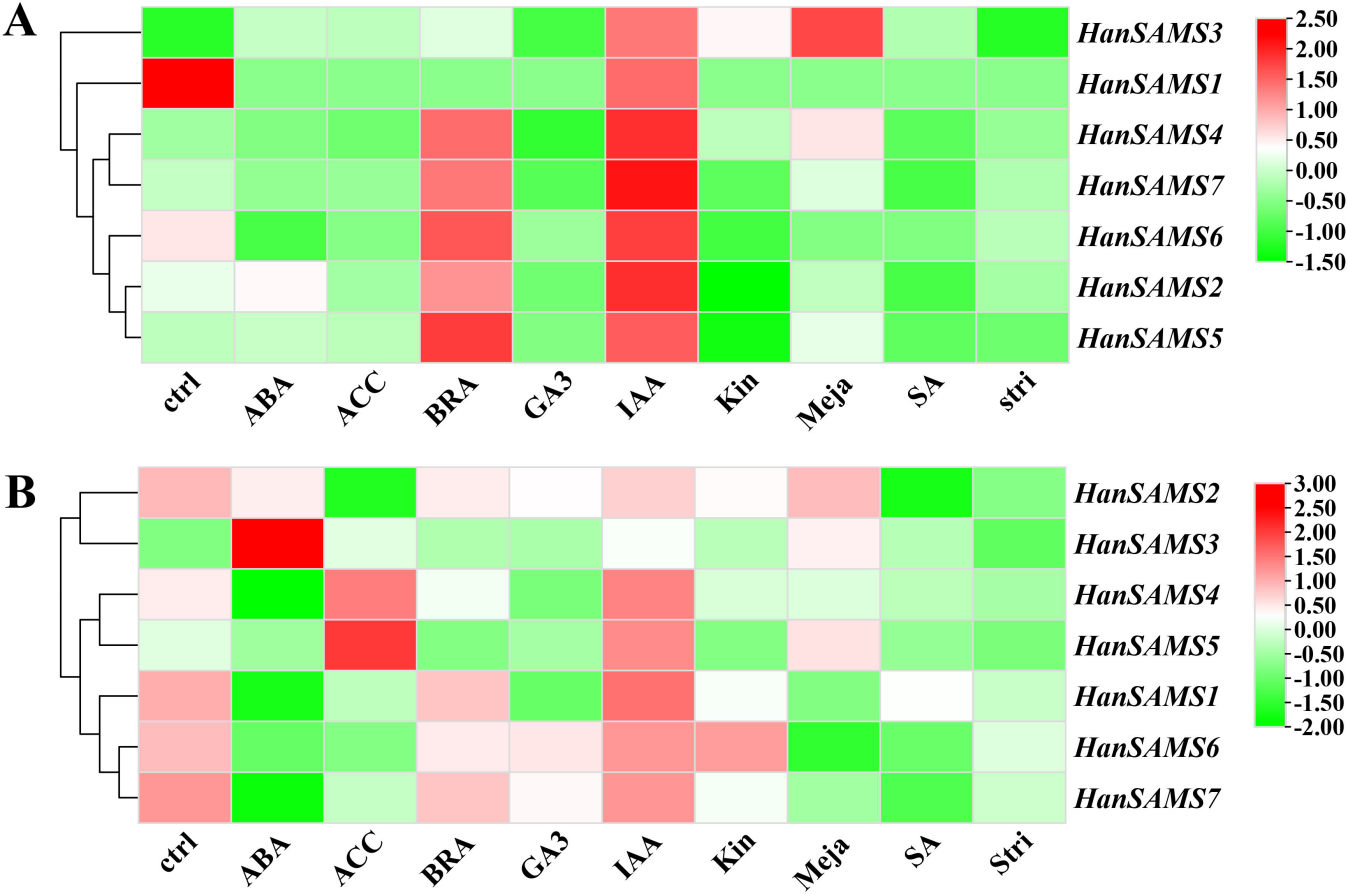
The expression of *HanSAMS* in different tissues and under different abiotic stresses. **(A)** *HanSAMS* gene expression in leaves under exogenous hormone treatment (SRP092742); **(B)** HanSAMS gene expression in roots under exogenous hormone treatment (SRP092742). ctrl, control; ABA, abscisic acid; ACC, Ethylene; BRA, Brassinosteroids; GA3, Gibberellic Acid 3; IAA, Indole Acetic Acid; Kin, Kinetin; Meja, Methyl-Jasmonate; SA, Salicylic acid; Stri, Strigolactone.

Promoter analysis revealed there are many hormonal response elements (**Figure 7A**) the RNA-seq data available for cultivated sunflower (XRQ) were examined and to elucidate the expression patterns of *SAMS* genes in response to hormones. A responsive pattern was observed across all *HanSAMS* genes upon IAA treatment in both leaves and roots (**Figure 9**), suggesting a pronounced sensitivity to auxin signaling (Song et al., 2024a). In leaf tissues, the *HanSAMS* gene family—excluding *HanSAMS1*—demonstrated a significant upregulation in expression following BRA (brassinosteroids) treatment, as depicted in **Figure 9A**. This underscores their crucial role in the regulatory pathways activated by BRA. However, in root tissues, all *HanSAMS* genes under BRA treatment conditions did not exhibit a significant increase compared to the control samples (**Figure 9B**). For other different hormone treatments, we also observed the different expression patterns in different tissues. For instance, *HanSAMS3* demonstrates its highest expression levels in leaves following MeJA treatment (**Figure 9A**), whereas in roots, the ABA treatment elicits its peak expression (**Figure 9B**). These results indicate that the *HanSAMS* gene expression is modulated in a tissue-specific manner in response to hormonal signals.

### 3.8 Expression patterns of *HanSAMS* genes under drought and salt stresse

**Figure 10:**
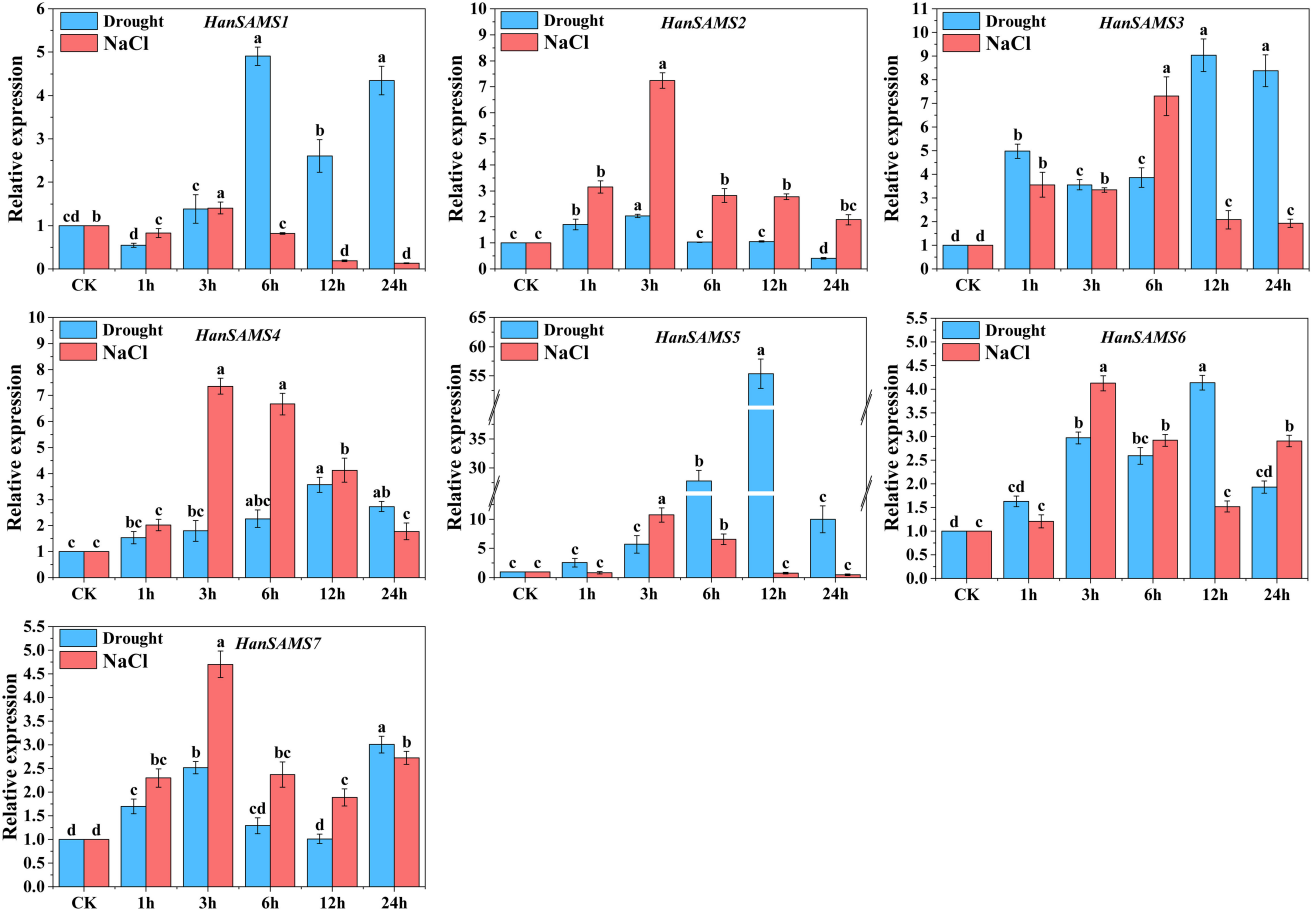
Expression patterns of *HanSAMS* genes under drought and NaCl stress treatments. a, b, c, d bar indicates a significant difference between the experimental treatments and control (CK) treatment (Significant differences were determined using the Duncan’s method of univariate ANOVA with a significance level of *P <0.05*).

Considering that the *cis*-elements responding to various stress existed in the promoter sequences of *HanSAMS* genes (**Figure 7A**), we conducted a quantitative analysis of the *HanSAMS* gene using qRT-PCR to examine their expression profiles under drought and salt stresses (**Figure 10**). Our findings revealed that the *HanSAMS* genes exhibited distinct expression patterns at various time intervals (0 h, 1 h, 3 h, 6 h, 12 h, 24 h) following exposure to drought and salt stresses. All of the *HanSAMS* genes showed increased expression levels at different times under stresses, and some differences were extremely significant when compared with the untreated group (CK, 0h). In the case of drought treatment, four of the seven *HanSAM* genes, including *HanSAMS3*, *HanSAMS4*, *HanSAMS5* and *HanSAMS6*, showed the highest upregulation at 12th hour, while *HanSAMS1* showed the highest upregulation at the 6th hour. Notably, we observed that the expression level of the *HanSAMS5* gene under drought stress is the highest among all *SAMS* genes (exceeding 55-fold at the 12th hour). In the case of salt treatment, five of the seven *HanSAM* genes, including *HanSAMS2*, *HanSAMS4*, *HanSAMS5*, *HanSAMS6* and *HanSAMS7*, showed the highest upregulation at the 3th hours and gradually downregulated thereafter, while *HanSAMS3* was up-regulated to highest point at 6 th of treatment. Notably, we also observed that the expression level of the *HanSAMS5* gene under salt stress is the highest among all *SAMS* genes (exceeding 10-fold at the 3th hour). In summary, most of *HanSAMS* genes exhibit responsiveness to both salt and drought stress treatments, with a more rapid response observed for salt stress (peak at 3th hour) compared to drought stress (peak at 12th hour).

## 4 Discussion

S-adenosylmethionine (SAM) is produced through the catalysis of methionine and adenosine triphosphate (ATP) by the enzyme S-adenosylmethionine synthetase (*SAMS*) (Fontecave et al., 2004). *SAM* genes play a crucial role in various cellular pathways, including those associated with ethylene and polyamine biosynthesis, methionine metabolism, as well as transmethylation and transsulfuration processes (Chen et al., 2016; Sauter et al., 2013). In the current study, *SAMS* genes have been analyzed by an extensive use of bioinformatics, such as *Arabidopsis* (4), *rice* (3), *tomato* (4), *Eggplant* (4), *Triticum urartu* (3), *Barley* (4), *Sorghum* (3), *Medicago truncatula* (5), *Soybean* (9) (Heidari et al., 2020). In this study, 7 *XRQ-HanSAMS*, 6 *HA89-HanSAMS*, 7 *HA300-HanSAMS*, 6 *IR-HanSAMS*, 7 *LR1-HanSAMS*, 6 *PI659440-HanSAMS*, 6 *PSC8-HanSAMS*, 6 *OQP8-HanSAMS*, and 7 *RHA438-HanSAMS* genes were identified in nine sunflower genomes, respectively. The phylogenetic analysis of the *HanSAMS* genes were performed to examine their relationships, the results indicated that they could be divided into seven groups (SAMS1-SAMS7) (**Figure 1**). It is not the case that every group encompasses all species. Only SAMS1 and SAMS2 are present in all nine varieties, while SAMS3 is found in only seven, suggesting the genetic diversity among different cultivars. The gene structure analysis revealed that all the 58 *HanSAMS* genes were intron-less and contain only one exon (**Figure 4A**), which is consistent with the results of previous studies in other species (Sun et al., 2022; Kilwake et al., 2023). Furthermore, the *cis*-elements analysis in the promoter region of the *HanSAMS* genes indicated that they might be primarily involved in the plant hormonal signals, light, and abiotic stresses responsiveness (**Figure 7**), this is similar to the findings of *cis*-elements in plants such as *Arabidopsis* and *Triticum aestivum* (Cheng et al., 2012; Shen et al., 2003). Our findings also suggest that one or two cultivars show a number notably different with other cultivars while most of *HanSAMS* genes within the same group share similar type and number of regulatory elements across cultivars, The results of the Ka/Ks analysis indicate that the *SAMS* gene family in nine sunflowers has predominantly experienced purifying selection throughout its evolutionary history.

Codon bias plays a complex role in the formation of gene mutation and the results of selection, but it is also important for the structure, function and expression of genes encoding proteins that are closely linked, and affects evolution (Chen et al., 2004; Hartl et al., 1994; Hershberg and Petrov, 2008). The codon usage bias of sunflower *SAMS g*ene families in nine cultivated species, ENc-plot, PR2-plot and neutrality curve analysis indicated that codon usage bias formation of sunflower *SAMS* gene families may be the result of base mutations, natural selection and other factors. Through RSCU analysis, it was found that high frequency codons in sunflower *SAMS* gene families of nine cultivated species preferred G/C ending, and the codon with the largest RSCU value encodes Leucine (Leu, CUU), Glycine (Gly, GGU), and Arginine (Arg, AGG). The codon bias of the plant genome can be analyzed and studied by a correlation index, and the frequency of codon usage between species at the order and family level is different; thus, the genetic relationship between species can be analyzed by a correlation index (Puigbo et al., 2008; Chen et al., 2014; Wang et al., 2011).

Previous research has indicated that *SAMS* genes are often activated by a variety of hormonal treatments and abiotic stresses. For instance, *AtSAMS3* and *AtSAMS4* are upregulated under biotic stress and brassinosteroid (BR) treatment, but downregulated in response to abiotic stresses such as salt, heat, and temperature stress, as well as ABA application (Heidari et al., 2020). In this study, we discovered that *HanSAMS* genes exhibit high expression levels in sunflower leaves when subjected to brassinolide (BRA) and indole-3-acetic acid (IAA) treatments, as revealed by previously published RNA-seq data (**Figure 9**). BRs are emerging as a plant hormone of significant importance due to their role in stress responses, including extreme temperatures and drought (Brewer et al., 2013; Ha et al., 2014; Nolan et al., 2020). Recent studies have shown that the overexpression of BRL3, a vascular BR receptor, enhances drought responses without hindering growth in *Arabidopsis* (Fàbregas et al., 2018). ABA and IAA are known as a hormone responsive to abiotic stresses such as drought, heat, low temperature, radiation and salt stress (Vishwakarma et al., 2017). However, in root tissues, all *HanSAMS* genes exhibiting reduced expression under BRA and IAA treatment conditions (**Figure 9**), indicating that the *HanSAMS* gene expression is modulated in a tissue-specific manner in response to hormonal signals.

In our promoter analysis, a multitude of MYB-related elements were identified in *HanSAMS* genes (**Figure 7**), Several studies have highlighted MYB as a crucial transcription factor associated with plant drought resistance and a key player in the transcriptional regulatory network governing plant responses to drought and salt stress (Espartero et al., 1994; Baldoni and Genga, 2015; Leng and Zhao, 2020; Sun et al., 2022). The overexpression of the *SAMS* gene from *Lycoris radiata* in *E. coli* has been shown to enhance plant tolerance to salt stress (Li et al., 2013). Given the presence of drought and salt responsive *cis*-elements in the promoter regions of *HanSAMS* genes, we performed a qRT-PCR analysis to assess all 7 *XQR-HanSAMS* expression dynamics under drought and salt stress conditions (**Figure 10**). Our results indicated that *HanSAMS* genes displayed unique expression profiles at different time points following stress exposure. Notably, *HanSAMS5* showed the most significant upregulation under both stress types, with over 55-fold increase at the 12th hour for drought and over 10-fold at the 3th hour for salt stress. This suggests that *HanSAMS5* may play a crucial role in the plant’s response to adverse environmental conditions.

## 5 Conclusion

The study identified 58 *HanSAMS* genes in nine sunflowers through whole-genome bioinformatics analysis. The identified *HanSAMS* genes are distributed across seven chromosomes, exhibiting a conserved exon-intron structure devoid of introns. Phylogenetic analysis has uncovered that the sunflower SAMS genes have expanded due to recent WGT-1 and WGD-2 events, resulting in three homologous branches, each comprising two discrete SAMS groups. The analysis of codon usage bias revealed a pronounced preference for high-frequency codons ending in G or C, notably those encoding glycine, leucine, and arginine, highlighting the significant role of natural selection in shaping the evolution of the *HanSAMS g*enes. A detailed promoter analysis revealed a wealth of stress-responsive *cis*-elements, suggesting their regulatory roles in stress tolerance. Moreover, expression profiling under hormonal stimuli and abiotic stresses, especially the marked upregulation of *HanSAMS5*, points to its pivotal role in managing multiple abiotic stresses. Collectively, these findings provide valuable insights into the functional diversity and evolutionary dynamics of the *SAMS* genes in sunflowers, laying a robust foundation for future research aimed at enhancing sunflower stress resilience through genetic improvement strategies.

## Data Availability

The original contributions presented in the study are included in the article/**Supplementary Material**. Further inquiries can be directed to the corresponding author.
