# Supplementary material for: Pan-genome wide identification and analysis of the SAMS gene family in sunflowers (Helianthus annuus L.) revealed their intraspecies diversity and potential roles in abiotic stress tolerance
Source: Front Plant Sci. 2024 Nov 13;15:1499024. doi: 10.3389/fpls.2024.1499024 (PMC11598334; doi:10.3389/fpls.2024.1499024)
Supplement: Supplementary file 2 [file Image1.pdf]

Figure S1-Figure S5

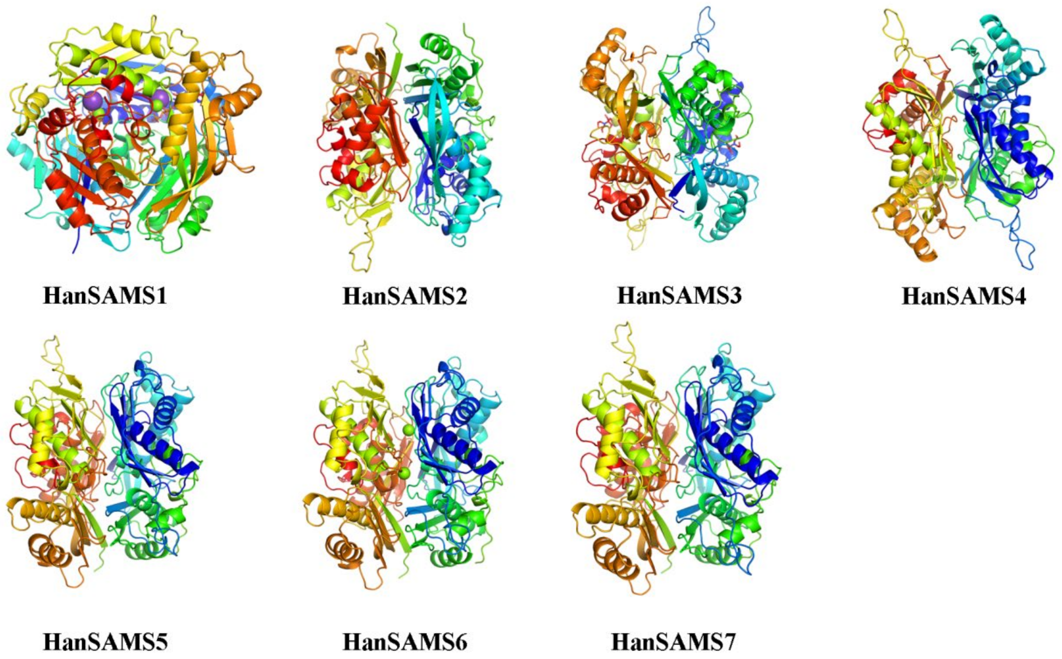

Figure S1 The tertiary structure prediction of HanSAMS proteins in XRQ cultivated sunflower.

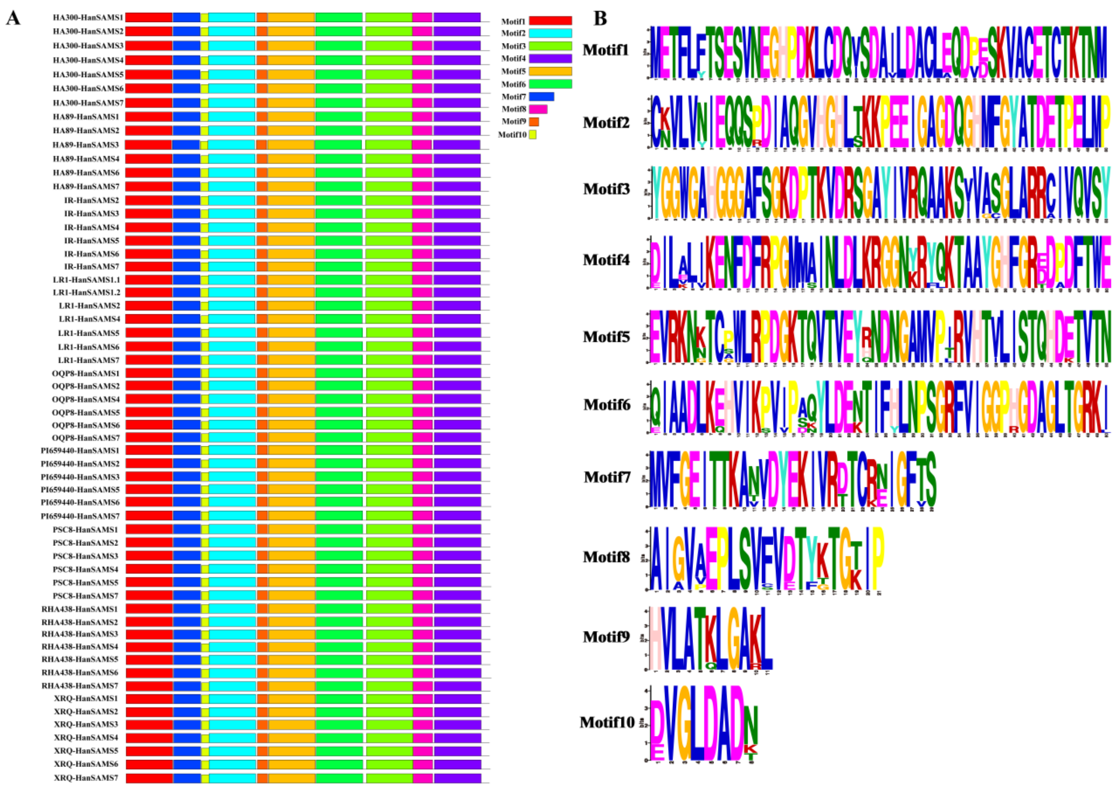

Figure S2 Sequence conservation analysis of HanSAMS proteins. (A) Conserved motif distribution of SAMS proteins in nine cultivated sunflowers. (B) Ten conserved motifs. The motif2 and motif5 were s-adenosylmethionine synthase domain (central domain), motif1 was s-adenosylmethionine synthase domain (N-terminal domain), motif3 and motif4 were s-adenosylmethionine synthase domain (C-terminal domain), about 50 amino acid residues long and is considered a key element.

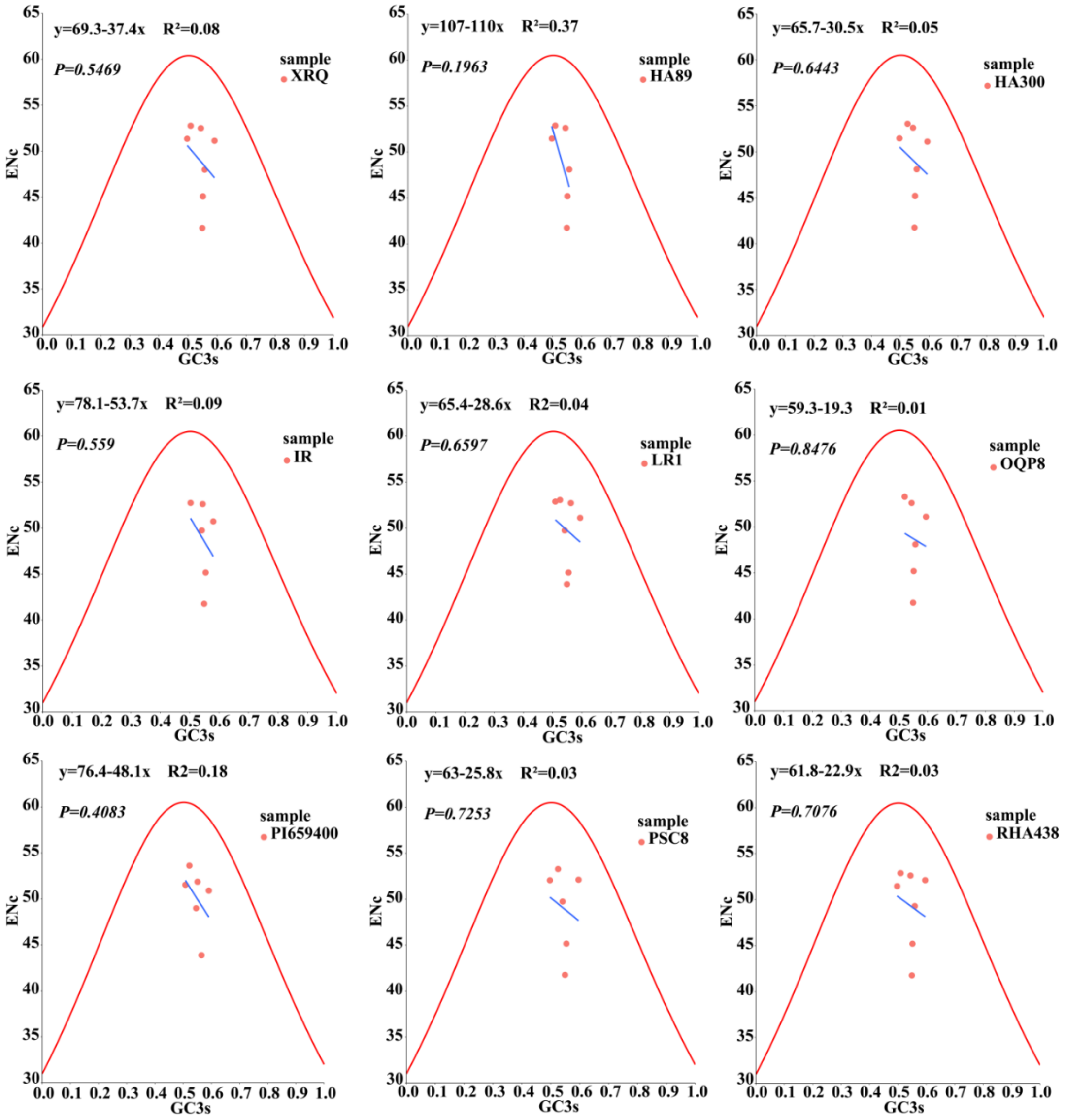

Figure S3 ENc-plot analysis of the *SAMS* gene family in nine sunflowers.

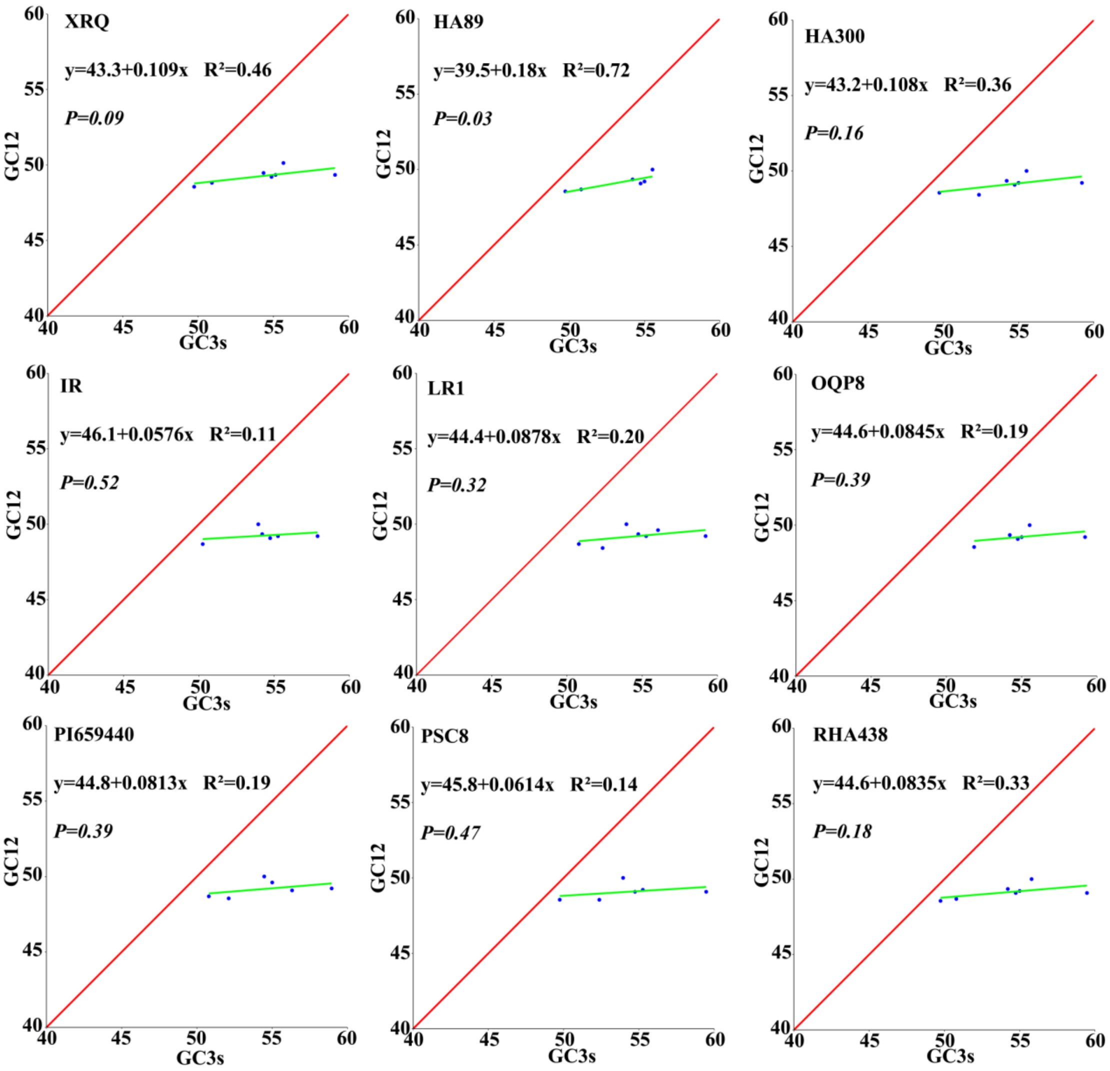

Figure S4 Neutrality curve analysis of the *SAMS* gene family in nine sunflowers.

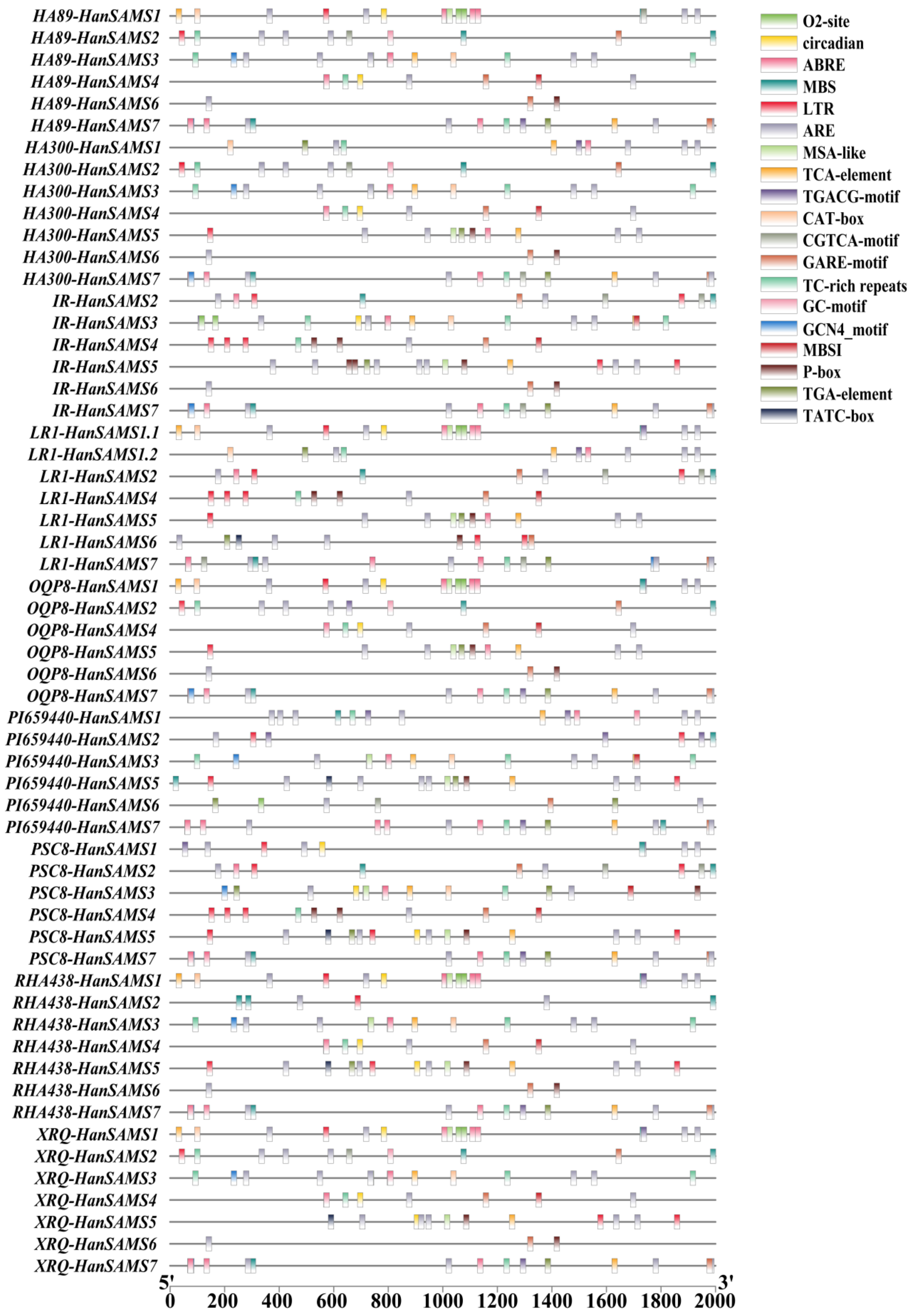

Figure S5 Predictive analysis of cis-acting regulatory elements in the promoter region of *SAMS* genes in nine sunflowers.
